# Supplementary material for: Dynamic Mode Decomposition of Multiphoton and Stimulated Emission Depletion Microscopy Data for Analysis of Fluorescent Probes in Cellular Membranes
Source: Sensors (Basel). 2024 Mar 25;24(7):2096. doi: 10.3390/s24072096 (PMC11013970; doi:10.3390/s24072096)
Supplement: Supplementary file 1 [file sensors-24-02096-s001.zip › sensors-2899868-supplementary.pdf]

sensors

MDPI

Supporting information for

Dynamic mode decomposition of multiphoton and stimulated emission depletion microscopy data for analysis of fluorescent probes in cellular membranes

Daniel Wüstner [1,*], Jacob Marcus Egebjerg [1] and Line Lauritsen [1]

[1] Department of Biochemistry and Molecular Biology University of Southern Denmark, DK-5230 Odense M, Denmark Affiliation
* Correspondence: wuestner@bmb.sdu.dk

Citation: To be added by editorial staff during production.

Academic Editor: Firstname Lastname

Received: date
Revised: date
Accepted: date
Published: date

Copyright: © 2024by the authors. Submitted for possible open access publication under the terms and conditions of the Creative Commons Attribution (CC BY) license (https://creativecommons.org/licenses/by/4.0/).

Dynamic modes and singular values of 2D MP sequences of DHE

In the absence of cellular displacements, 2D MP image sequences consist of a simple singular value spectrum resulting in a single dynamic mode with zero imaginary contribution, as shown in Figure S1, below.

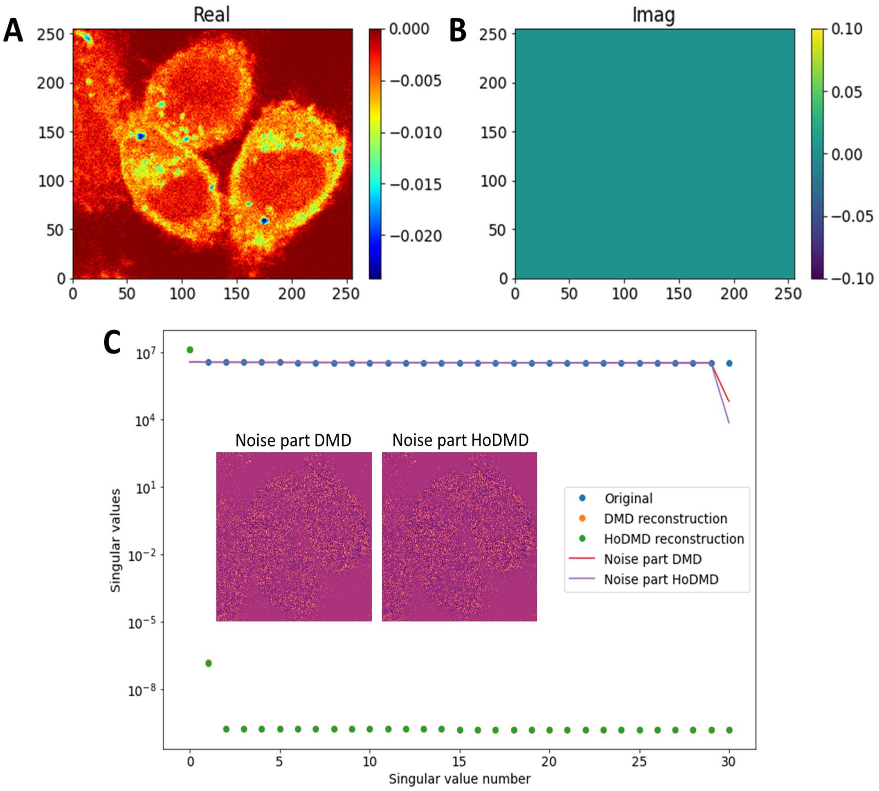

Figure S1. DMD and singular value spectrum for the 2D MP sequence of DHE shown in Figure 2. Only one dynamic mode with non-zero real part (**A**) but zero imaginary contribution(**B**) was identified by standard DMD. There is only one dominant singular value, σ_1, see first green dot in (**C**). The remaining singular values, σ_i with i >1, are constant and lie much lower for the DMD and HoDMD reconstruction compared to the raw data (**C**). Subtracting the reconstructed from the original image stack gives a noise dominated image for each method (inset in **C**). The singular value spectrum of those noise images is flat and coincides precisely with the singular values σ_i with i >1 of the raw image data (compare blue dots and red and violet line for σ_i with i >1). Thus, the low-rank approximation of the Koopman matrix A, which is part of the DMD and HoDMD algorithms, is very efficient in removing Poisson-distributed shot noise from MP microscopy data.

Reconstruction of point spread function by DMD and HoDMD

A theoretical PSF based on the Born & Wolf model with a numerical aperture (NA) of 1.4, a pixel size of 100 nm and a sampling distance of Δz = 250 nm was simulated with a wavelength of 610 nm using the ImageJ plugin 'PSF generator' available at (https://big-www.epfl.ch/algorithms/psfgenerator/) [1]. The PSF was analyzed by either standard DMD or HoDMD, the latter with different extends of z-shifts (i.e., d=6 or 12), which gave identical results.

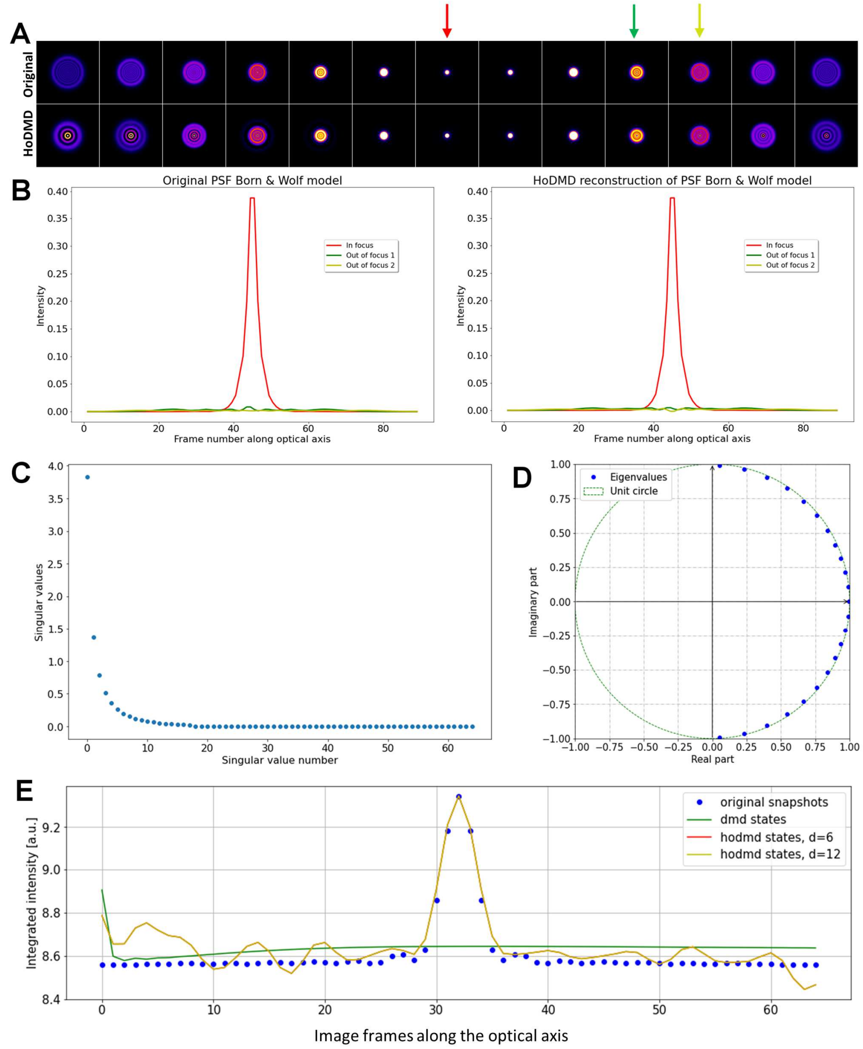

Figure S2. DMD and HoDMD of a theoretical PSF model of a microscope. Montage of images along the optical axis for the simulated (upper row) and HoDMD reconstructed PSF (lower row) is shown in (**A**). Arrows indicate selected frames for which 1D intensity profiles are shown in (**B**). Left panel shows line profiles for the original PSF and right panel for the HoDMD-reconstructed PSF (in-focus profile in red, and two out-of-focus line profiles in green and yellow, respectively). Singular values for the 3D stack of the simulated PSF is shown in (**C**). Eigenvalues for HoDMD reconstruction of this PSF are plotted on the unit circle (**D**). Integrated intensity along the optical (z-) axis for the original data (blue dots), the DMD reconstruction (green line), and the HoDMD reconstruction with d=6 (red line) or d=12 (yellow line) are shown in (**E**).

Effect of image convolution and noise on DMD/HoDMD performance

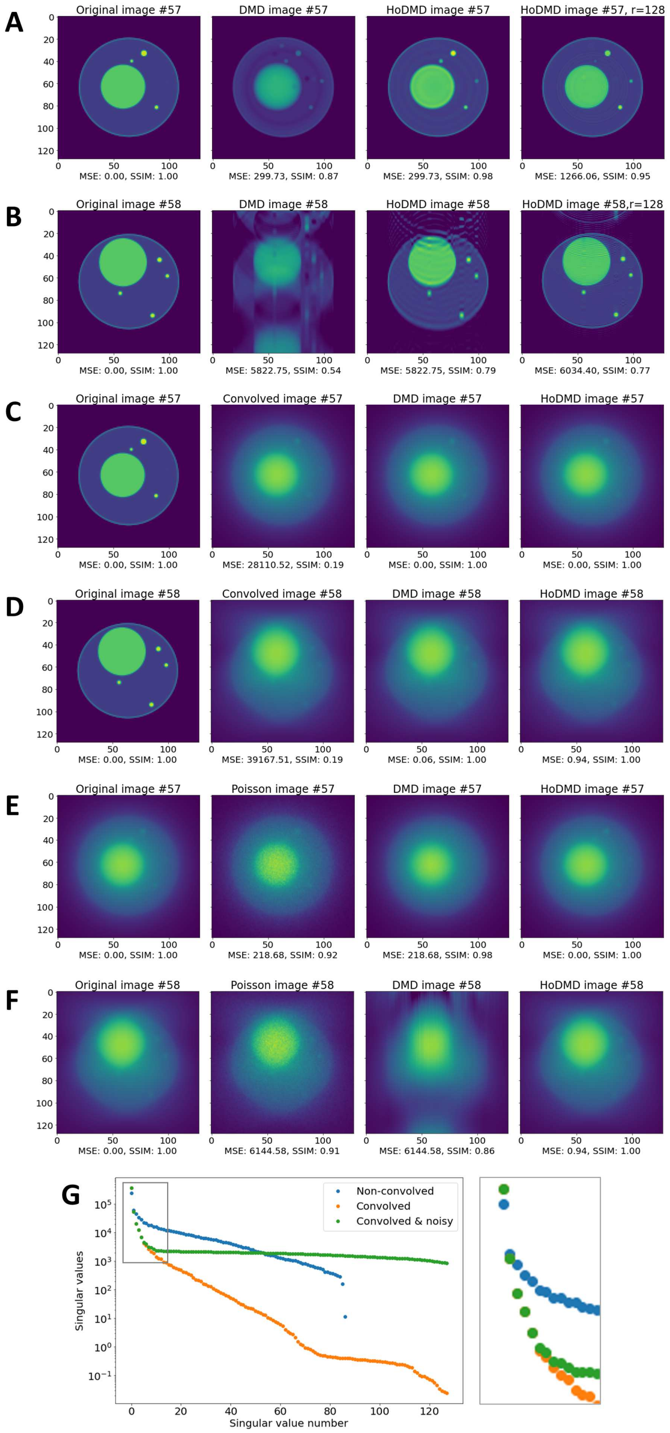

Figure S3. A 3D cell phantom was generated and reconstructed using DMD or HoDMD. Results are shown prior to (**A** and **B**) or after 3D convolution with a theoretical PSF (**C** to **F**). Poisson noise is added to the convolved stacks in **E** and **F**). xy-views of frame 57 out of 128 are shown in **A, C** and **E**, while xz-views along line 58 are shown in **B**, **D** and **F**. The singular value spectrum of the 3D data is shown on a semilogarithmic plot in (**G**). The singular values decay more rapidly after convolution but plateau in the presence of noise. The inset in the rectangular box is enlarged as zoomed version to the right and shows the largest singular values for all three conditions.

Dynamic modes determined by DMD and HoDMD for polarimetry data

Two-photon polarimetry data of TF-Chol was analyzed by DMD and HoDMD, as described in Figure 5 and accompanying text. Decomposition of the progression between snapshots, i.e., images representing the fluorescence response to the rotating incident light vector is shown for both methods.

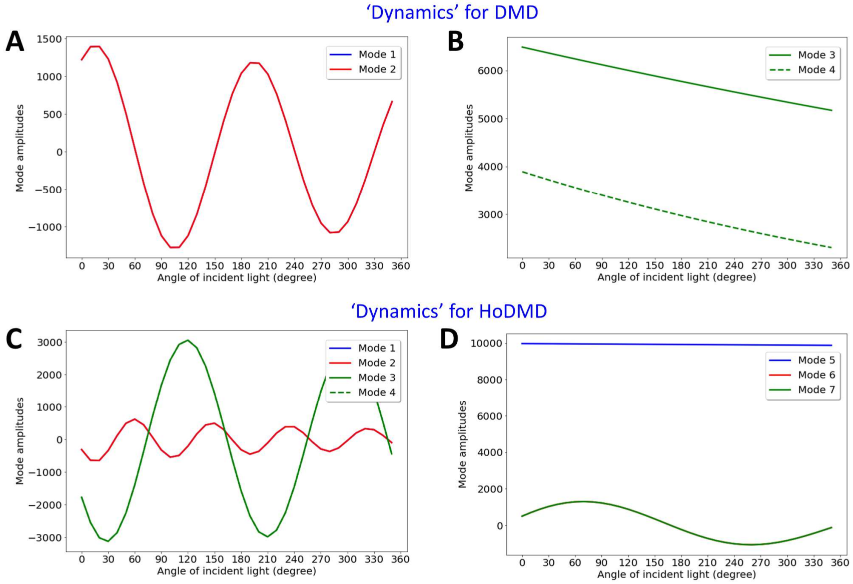

Figure S4. Progression of dynamic modes 'dynamics' of two-photon orientation of TF-Chol in cell membranes as function of orientation angle of the incident two-photon laser light. Standard DMD identified four modes, two with oscillating real part (**A**) and two modes with exponentially decaying real part (**B**). HoDMD recovered seven modes, of which the real part of four are shown in (**C**) and that of the three remaining in (**D**).

Singular value spectrum of 3D STED data

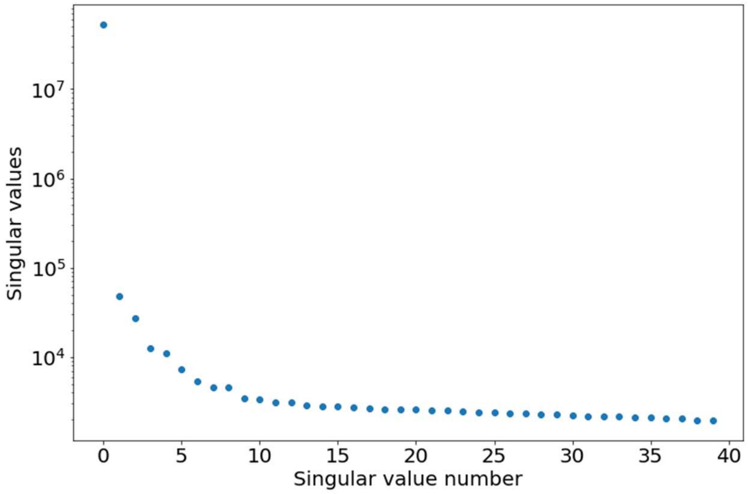

Figure S5. The singular value spectrum of the 3D STED data is shown on a semilogarithmic plot. Clearly, the singular values decay rapidly, such that only few dynamic modes are needed to describe the data. See main text for further explanations.

Dynamic modes and eigenvalues of 3D STED image stacks of Nile Red

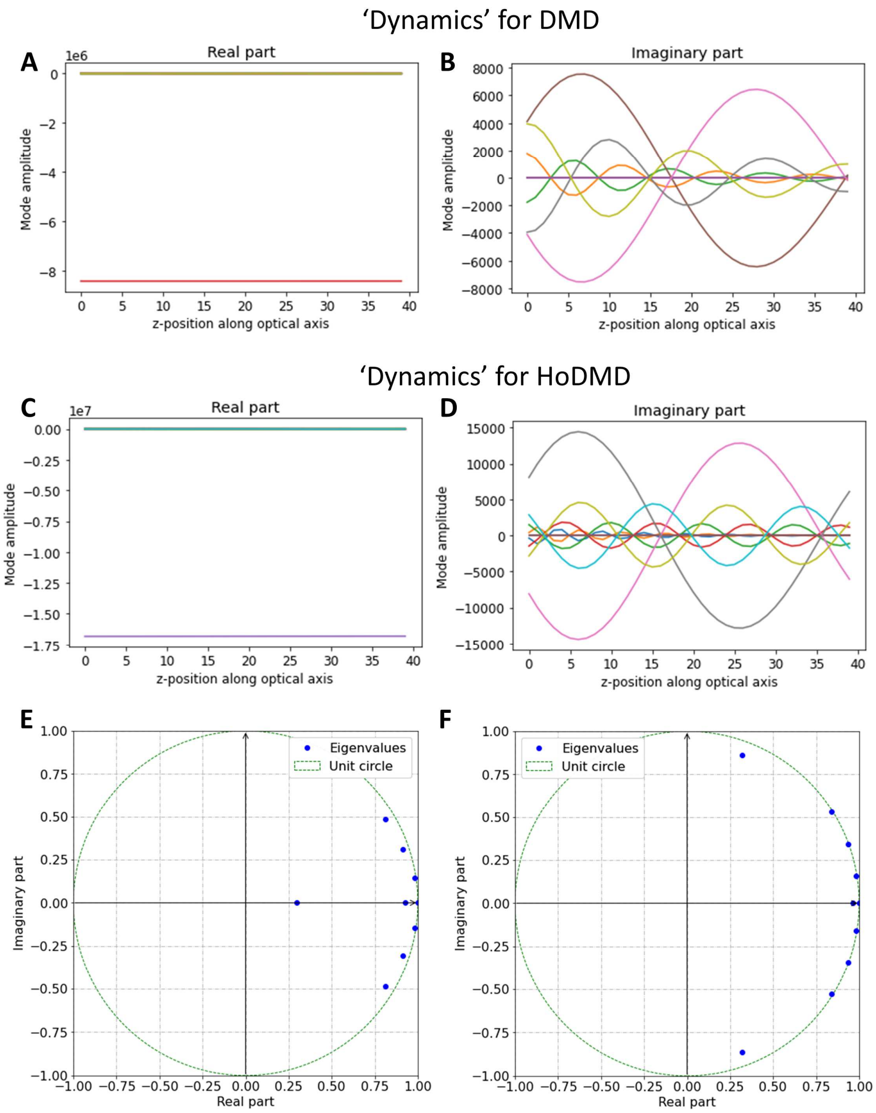

Figure S6. Progression of dynamic modes ‘dynamics’ of 3D-STED microscopy of Nile Red in cell membranes as function of z-position along the optical axis. Standard DMD identified nine modes, three with only monotonous real part (**A**) and seven modes with periodically changing imaginary part (**B**). HoDMD recovered ten modes, of which two with only the real part (**C**) and eight with non-zero real and imaginary part. The imaginary part of the latter is shown in (**D**). Eigenvalues for DMD (**E**) and for the HoDMD reconstruction of this data (**F**) are plotted on the unit circle.

References

[1] H. Kirshner, F. Aguet, D. Sage, M. Unser, 3-D PSF fitting for fluorescence microscopy: implementation and localization application, Journal of microscopy, 249 (2013) 13-25.
